# Shikonin alleviates asthma phenotypes in mice via an airway epithelial STAT3-dependent mechanism

**DOI:** 10.1515/med-2024-1016

**Published:** 2024-10-21

**Authors:** Yao Zhang, Lizhan Chen, Haifeng Ouyang

**Affiliations:** Department of Respiratory Medicine, Xi’an International Medical Center Hospital, Xi’an, 710101, China; Department of Respiratory Medicine, Xi’an International Medical Center Hospital, No. 777 Xitai Road, Xi’an, 710101, China

**Keywords:** shikonin, asthma, STAT3, Th1/Th2, Th17/Treg

## Abstract

**Background:**

Asthma is an inflammatory disease where the balance between Th1/Th2 and Th17/Treg plays a crucial role in its pathogenesis. Shikonin is used to treat a variety of autoimmune diseases due to its good anti-inflammatory activity. However, the effect and mechanism of shikonin on asthma remain unknown.

**Method:**

Mice were sensitized with ovalbumin (OVA)/house dust mite (HDM) and treated with shikonin. Lung inflammation was assessed histologically and via flow cytometry. Bronchoalveolar lavage fluid (BALF) was analyzed for cell counts and cytokines. Shikonin’s impact on p-STAT3 was studied *in vivo* and *in vitro.*

**Results:**

Shikonin inhibited OVA or HDM-induced inflammation and airway hyperresponsiveness. Upon treatment, a restoration of the Th1/Th2 and Th17/Treg balance was observed, evidenced by a reduction in IL-4 and IL-17A levels in BALF, alongside an elevation in interferon-gamma and IL-10. Furthermore, shikonin impeded the infiltration of eosinophils, neutrophils, macrophages, and lymphocytes into lung tissue. The observed decrease in STAT3 phosphorylation and diminished nuclear translocation of p-STAT3 confirmed that shikonin promotes the balance of Th1/Th2 and Th17/Treg by regulating airway epithelial STAT3.

**Conclusion:**

Shikonin mitigates asthma symptoms through a STAT3-dependent mechanism, indicating its potential as an anti-asthmatic therapeutic agent.

## 1 Introduction

As a common chronic respiratory disease asthma with high morbidity and mortality is seriously affecting the health of people of all ages [1]. It is characterized by reversible airway obstruction, mucus hypersecretion, airway inflammation, and airway hyperresponsiveness, which often lead to wheezing, chest tightness, and breathlessness in asthmatic patients [2]. There is no gold standard for the diagnosis of asthma, because it is a heterogeneous disease with complex pathogenesis, and the clinical manifestations, treatment response, and prognosis of patients are different [3]. At present, the goal of treating asthma is to control the occurrence of asthma as much as possible, restore the lung function of patients, and reduce the risk of death [4]. In recent years, although glucocorticoids, bronchodilators, and some targeted drugs have been widely used in clinical practice, some patients cannot achieve complete control, and the incidence of asthma is still increasing [5]. Therefore, it is urgent to elucidate the pathogenesis of asthma and find effective therapeutic drugs.

Asthma is an inflammatory disease with an abnormal immune response, which is closely related to the activation of CD4^+^ T helper (Th) cells in the lung. Excessive immune response in asthmatic patients increases vascular permeability and causes airway inflammation, which is the result of the interaction between epithelial cells and immune cells [6]. In the process of immune response, there is an imbalance between Th1 and Th2. The highly expressed Th2 cells can stimulate type 2 immunity by secreting a large number of cytokines such as IL-4, IL-5, IL-9, and IL-13, which will lead to the accumulation of mast cells, macrophages, and eosinophils [7]. Increased airway responsiveness and airway inflammation are considered to be one of the pathogenesis of asthma [8]. IL-4 is a characteristic cytokine of Th2 cells as well as a major cytokine leading to asthma. On the one hand, by inducing the expression of mucin genes, IL-4 can lead to the excessive secretion of mucus by goblet cells within the airways [9]. On the other hand, IL-4 is also a factor secreted by T cells themselves, which can induce the occurrence of asthma by promoting the transformation of T cells into Th2 cells [10]. On the contrary, Th1 cells can release IL-2, IL-12, and interferon-gamma (IFN-γ) [11], among which the characteristic cytokine IFN-γ plays an inhibitory role in asthma by hindering the aggregation of eosinophils and reducing the expression of immunoglobulin [12]. In addition to Th1/Th2, Th17/Treg is important for the pathogenesis of asthma [13]. Immunosuppression can be mediated by Treg via secreting anti-inflammatory cytokines such as IL-10 and transforming growth factor-β1 [14]. However, inflammatory response can be induced by Th17 via generating IL-17A to promote the mobilization, recruitment, and activation of neutrophilic granulocyte [15]. Therefore, there is an imbalance between Th1/Th2 and Th17/Treg in asthma. The severity of asthma can be assessed by detecting the balance of Th1/Th2 and Th17/Treg.

Shikonin, the main bioactive component of lithospermum erythrorhizon roots has various biological functions such as anti-cancer, anti-inflammation, and promoting wound healing [16]. Studies have shown that shikonin can inhibit the inflammatory process of osteoarthritis by activating the mitogen-activated protein kinases (MAPK) and inhibiting the phosphorylation of STAT3 to down-regulate IL-1β-induced inflammatory factors in chondrocytes [17]. In rheumatoid arthritis, the inhibitory effect of lipopolysaccharide (LPS) on IL-10 expression in fibroblast-like synoviocytes can be significantly reversed by shikonin due to the inactivation of the PKC-NF-κB pathway, thereby reducing the production of tumor necrosis factor-α (TNF-α) [18]. Treatment of LPS-induced human primary nucleus pulposus cells with shikonin could reverse the expression levels of inflammatory cytokines (TNF-α and IL-1β) and apoptosis-related molecules (Bax, Bcl-2, and cleaved caspase 3), because shikonin can inhibit the expression of p-p65 and the nuclear translocation of p65. This study demonstrates that the anti-inflammatory and anti-apoptotic effects of shikonin on human intervertebral disc degeneration are achieved through the NF-κB pathway [19]. In addition, shikonin can reduce the secretion of pro-inflammatory factors such as TNF-α, IL-1, and IL-6 by inhibiting the polarization of M1 macrophages induced by LPS- and Th1-derived cytokines (such as IFN-γ and IL-1β), thereby alleviating collagen-induced arthritis in mice [20]. However, only a few studies have shown the effect of shikonin on inflammation in asthma mice. Wang et al. found that intraperitoneal injection of shikonin can reduce pathological airway remodeling, inflammatory cell infiltration, and collagen deposition in ovalbumin (OVA)-induced asthma mice. Further mechanism studies have shown that shikonin can inactivate NF-κB by blocking the activation of IκBα, which inhibits the proliferation and migration of primary airway smooth muscle cells and the activity of matrix metalloproteinases (MMP2 and MMP9), thereby reducing allergic airway remodeling [21]. Although the effect of shikonin on asthma has been reported, the mechanism by which shikonin inhibits inflammation in asthma mice has not been determined.

Asthma is mainly caused by airway inflammation, and STAT3 involved in inflammatory and immune response plays an important role in the pathogenesis of asthma [22]. A study in mice showed that lung inflammation can be caused by STAT3 via accelerating the development of Th17 cells and promoting the production of cytokines by Th2 and Th17 cells, which will lead to the occurrence of asthma [23]. Airway epithelial cells play an extremely important role in the pathogenesis of asthma. The specific knockout of STAT3 expressed in airway epithelial cells can significantly down-regulate house dust mite (HDM)-induced airway inflammation in asthma mice [24,25]. However, whether shikonin can inhibit asthma through this mechanism remains to be explored.

To evaluate the mechanism by which shikonin alleviates the phenotype of asthma mice, we used OVA- and HDM-induced asthma mice to observe the effects of shikonin on the expression of Th1-, Th2-, Treg-, and Th17-related cytokine profiles, airway responsiveness, and airway inflammation *in vivo*. In addition, we evaluated the effects of shikonin on the expression and localization of p-STAT3 in airway epithelial cells. *In vitro*, we analyzed whether the STAT3 activator colivelin could reverse the inhibitory effect of shikonin on asthma and detected the expression of p-STAT3 and its downstream factors.

## 2 Materials and methods

### 2.1 Drug and reagents

Shikonin (S7576, purity ≥98%) was purchased from Sigma (St. Louis, MO, USA). OVA (100 μg/mL, Sigma) was mixed with an equal volume of 10% (w/v) alum (Sigma) in distilled water. HDM (50,000 BU/mL) was obtained from Allergopharma (Reinbeck, Germany). STAT3 agonist (Colivelin) was acquired from Tocris (Bristol, Britain).

### 2.2 Mouse model of asthma

Female BALB/c mice (4 weeks old) were purchased from Vital River Laboratory Animal Technology Co., Ltd (Beijing, China) and raised in a constant temperature SPF environment. Mice were randomly divided into five groups: control, OVA model, HDM model, OVA + shikonin (4 mg/kg), and HDM + shikonin (4 mg/kg), with three mice in each group. For the OVA model group, mice were sensitized with an intraperitoneal injection of 50 μg OVA mixed with 10% aluminum hydroxide solution on days 1, 14, and 28. From the 21st day, the mice were placed in a closed container and atomized with 8 mL of OVA (1% in PBS) solution for 30 min, twice a week for 8 weeks. For the HDM model group, mice were sensitized through intraperitoneal injection of 20 μg of HDM combined with 1 mg of aluminum hydroxide constituted in 0.1 mL of saline on days 0 and 14. The mice were further challenged with intranasal instillation of 10 μg of HDM in 30 μL of saline three times per week from weeks 3 to 8. For OVA + shikonin (4 mg/kg) and HDM + shikonin (4 mg/kg) groups, mice in each group were treated with shikonin (4 mg/kg) by intratracheal instillation half an hour before each challenge. The control group was treated with normal saline instead of OVA, HDM, and shikonin. All animal procedures were performed with the approval of the Animal Care and Use Committee of Xi’an International Medical Center Hospital (Approval No. GJYX-KY-2023-006).

### 2.3 Cell culture and stimulation

The mouse lung epithelial-12 (MLE-12) cells were obtained from the Cell Bank of the Chinese Academy of Sciences (Shanghai, China) and cultured in DMEM/F12 (Gibco, USA) containing 2% fetal calf serum. The cells were incubated with TNF-α (20 ng/mL) for 2 h to induce the *in vitro* model of asthma. To investigate the effect and mechanism of shikonin, shikonin (5 μg/mL) and colivelin (25 μg/mL) were used to treat cells prior to TNF-α stimulation.

### 2.4 Airway hyperresponsiveness

After the last challenge, the mice in each group were inhaled with 300 μL of 50 mg/mL methacholine (Mech), and the atomization was monitored for 3 min each time. The Penh values reflecting airway responsiveness were recorded using a Buxco non-invasive pulmonary function detector (Wilmington, NC, USA).

### 2.5 Bronchoalveolar lavage fluid (BALF) cell count and serum analysis

BALF was collected by flushing the lungs of mice with 0.9 mL of cold PBS containing 2 mM ethylene diamine tetraacetic acid and 2% fetal bovine serum. After BALF was centrifuged at 1,500 × *g* for 10 min at 4°C, the cell precipitate was resuspended. Then, the total number of BALF cells was calculated using a hemocytometer, and the number of differential cells (eosinophils, neutrophils, macrophages, and lymphocytes) was determined by Wright-Giemsa staining. The enzyme linked immunosorbent assay (ELISA) kits (R&D systems, Minneapolis, MN, USA) were used to detect the levels of IFN-γ, IL-4, IL-17A, and IL-10 in BALF supernatant.

### 2.6 Lung histopathology

The mice were euthanized with isoflurane, and the left lung was fixed with 4% paraformaldehyde. After dehydration, embedding, and sectioning, the specimens were stained with hematoxylin/eosin (HE). The degree of inflammation was scored according to the infiltration of inflammatory cells around the airway: 0, no infiltrates; 1, few inflammatory cells; 2, one layer of inflammatory cells; 3, 2–4 layers of inflammatory cells; and 4, more than four layers of inflammatory cells.

### 2.7 Flow cytometry

The left lobe of the lung was digested to obtain single-cell suspension. Cells were stained with fluorescence-labeled antibodies (Abcam, Cambridge, UK) specific for APC-IFN-γ, PE-IL-4, PE-IL-17A, and PE-Foxp3. A CytoFLEX flow cytometer (Beckman Coulter, Villepinte, France) was used to detect the percentage of Th1 (CD4^+^ IFN-γ^+^), Treg (CD25^+^ FOXP3^+^), Th2 (CD4^+^ IL-4^+^), and Th17 (CD4^+^ IL-17^+^) cells.

### 2.8 Western blot analysis

Total protein was extracted with radio immunoprecipitation assay lysis buffer from airway epithelial cells or MLE-12 cells, and the concentration of these proteins was determined by bicinchoninic acid reagent. These proteins were separated by sodium dodecyl sulfate-polyacrylamide gel electrophoresis and transferred to a polyvinylidene fluoride membrane. Then, these membranes were blocked with 5% skim milk for 1 h and incubated with primary antibodies (Abcam) at 4°C overnight. The primary antibodies used in this study were GAPDH, anti-STAT3, anti-phospho (p)-STAT3, anti-IL-1β, and anti-IFN-γ. The next day, these membranes were washed three times with tris-borate-sodium tween and incubated with the horseradish peroxidase-conjugated secondary antibody for 1 h. Finally, the enhanced chemiluminescence substrate was used to display the bands, which were semi-quantitatively analyzed by the ImageJ software.

### 2.9 Immunofluorescence analysis

Airway epithelial cells or MLE-12 cells were seeded on glass slides, fixed with 4% paraformaldehyde, permeabilized with 0.5% Triton X-100, and blocked with 5% skim milk powder solution. At 4°C, these cells were incubated with primary antibodies against p-STAT3 (Abcam) overnight. On the second day, these cells were incubated with the secondary antibody in a dark room for 1 h. The nucleus was labeled with DAPI, and the fluorescence was observed under a fluorescence microscope.

### 2.10 ELISA

IL-1β kit (Abcam) and IFN-γ kit (Abcam) were used to detect the concentration of these factors in the culture supernatant of MLE-12 cells.

### 2.11 Statistical analysis

All experiments in this study were repeated three times, and the data were analyzed using GraphPad Prism 8.0 (GraphPad Software, Inc., La Jolla, CA, USA). Data are presented as mean ± standard error of the mean. One-way analysis of variance was used to compare the differences between groups. A *P* value of less than 0.05 is considered to be statistically significant.

## 3 Results

### 3.1 Shikonin alleviates the phenotypes of OVA- and HDM-induced asthma mice

**Figure 1:**
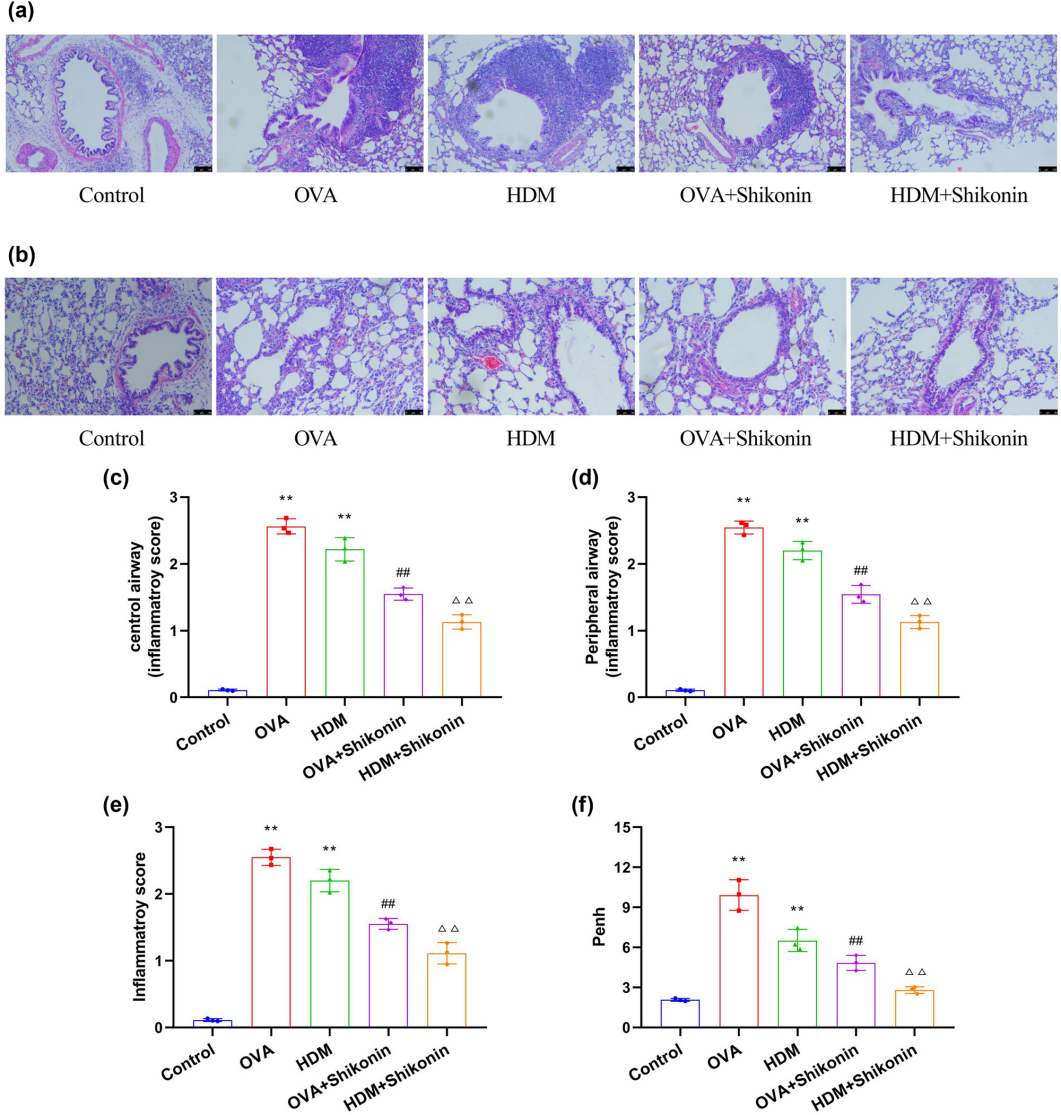
Shikonin alleviates the phenotypes of OVA- and HDM-induced asthma mice. (a) HE staining was used to detect the inflammatory infiltration of the central airway in the lung sections of mice. (b) HE staining was used to detect peripheral airway inflammatory infiltration in the lung sections of mice. (c) Central airway inflammation score. (d) Peripheral airway inflammation score. (e) Semi-quantitative score of inflammatory infiltration in the lung sections of mice. (f) The effect of shikonin on airway reactivity in asthma mice. Compared with the control group, **p < 0.01; compared with the OVA group, ^##^ *p* < 0.01; compared with the HDM group, ^△△^ *p* < 0.01, *n* = 3.

To investigate the effect of shikonin on the phenotypes of asthma mice, we examined airway inflammation and airway responsiveness of mice. First, we used OVA and HDM to sensitize and challenge 4-week-old BALB/c mice. At 7 weeks of age, asthma mice were treated by intratracheal instillation of shikonin (4 mg/kg) for 28 days. Then, HE staining was used to observe the inflammatory cell infiltration of the central and peripheral airway in the lung sections of mice. As shown in Figure 1a–e, the structure of alveolar tissue in the control group was normal, and no inflammatory cell infiltration was observed in the central and peripheral airway. The alveolar wall of OVA- and HDM-induced mice was significantly thickened, and the lumen was narrowed. The central and peripheral airways were infiltrated by a large number of inflammatory cells (lymphocytes, eosinophils, neutrophils, macrophages). After treatment with shikonin, the inflammation of lung tissue was improved. In addition, HE staining did not show significant goblet cell proliferation and staining of positive epithelial cells in the control group. In the OVA group and the HDM group, the necrosis of airway epithelial cells and the hyperplasia of goblet cells were obvious, and the function of airway mucus secretion was hyperactive. After treatment with shikonin, the positive cells of airway epithelium in the OVA + shikonin group and the HDM + shikonin group were less than those in the OVA and HDM groups by HE staining, and the mucus secretion of airway epithelium was significantly reduced. The airway responsiveness of the OVA group and the HDM group was higher than in the control group, while shikonin could decrease the airway responsiveness of asthma mice (Figure 1f). The above results collectively indicate that shikonin can reduce inflammation and airway responsiveness, thereby alleviating the phenotypes of asthma mice induced by OVA and HDM.

### 3.2 Shikonin induces CD4^+^ IFN-γ^+^ Th1 cells and CD25^+^ FOXP3^+^ Treg cells, and inhibits CD4^+^ IL-4^+^ Th2 cells and CD4^+^ IL-17^+^ Th17 cells

**Figure 2:**
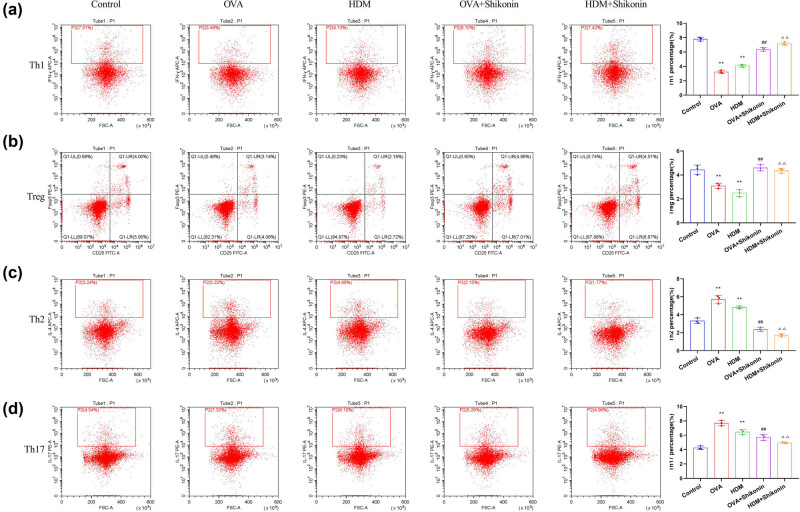
Shikonin alleviates the imbalance of Th1/Th2 and Th17/Treg in OVA- and HDM-induced asthma mice. Flow cytometry was used to evaluate the percentages of CD4^+^ IFN-γ^+^ Th1 cells (a), CD25^+^ FOXP3^+^ Treg cells (b), CD4^+^ IL-4^+^ Th2 cells (c), and CD4^+^ IL-17^+^ Th17 cells (d) in lung tissues. Compared with the control group, ^**^ *p* < 0.01; compared with the OVA group, ^##^ *p* < 0.01; compared with the HDM group, ^△△^ *p* < 0.01, *n* = 3.

To investigate the effect of shikonin on T-cell populations in asthma mice, we evaluated the phenotypes of CD4^+^ cells in lung tissues by analyzing the expression of IFN-γ, FOXP3, IL-4, and IL-17 via flow cytometry. Compared with the control group, OVA or HDM sensitization and challenge decreased the proportion of CD4^+^ IFN-γ^+^ Th1 cells and CD25^+^ FOXP3^+^ Treg cells (Figure 2a and d) and increased the proportion of CD4^+^ IL-4^+^ Th2 cells and CD4^+^ IL-17^+^ Th17 cells (Figure 2b and c). After treatment with shikonin, the proportion of Th1 and Treg cells was up-regulated (Figure 2a and d), and the number of Th2 and Th17 cells was down-regulated (Figure 2a and d). These data suggest that shikonin can alleviate the imbalance of Th1/Th2 and Th17/Treg by inducing CD4^+^ IFN-γ^+^ Th1 cells and CD25^+^ FOXP3^+^ Treg cells, and inhibiting CD4^+^ IL-4^+^ Th2 cells and CD4^+^ IL-17^+^ Th17 cells, thereby improving the inflammatory responses caused by OVA or HDM.

### 3.3 Shikonin inhibits the infiltration of immune cells and regulates the expression of Th-cell-related cytokines

**Figure 3:**
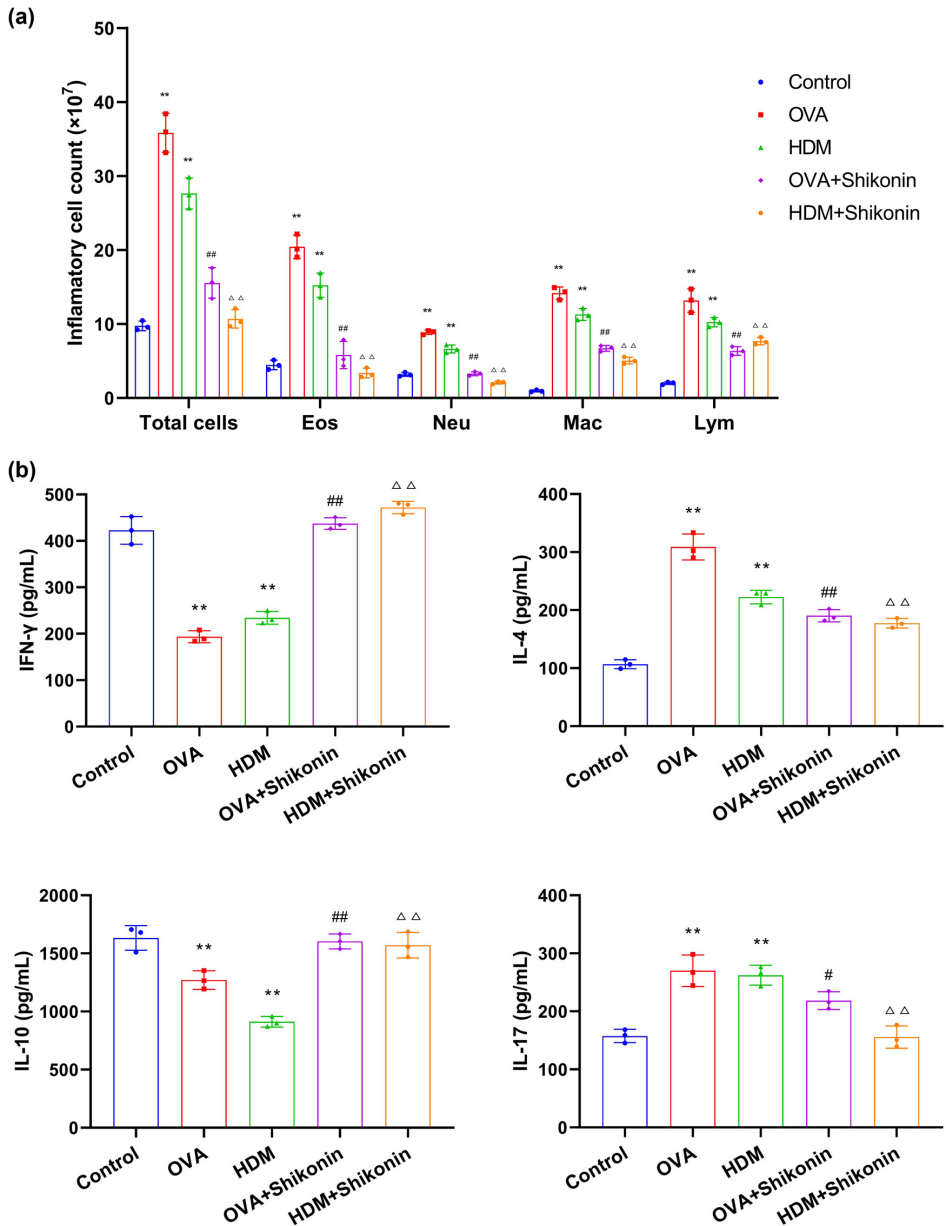
Shikonin alleviates immune cell infiltration and regulates the expression of Th cell-related cytokines. (a) The number of inflammatory cells, eosinophils (Eos), neutrophils (Neu), macrophages (Mac), and lymphocytes (Lym) in bronchoalveolar lavage fluid (BALF). (b) The concentration of Th cell-related inflammatory factors in BALF was detected by ELISA. Compared with the control group, ^**^ *p* < 0.01; compared with the OVA group, ^#^ *p* < 0.05; compared with the OVA group, ^##^ *p* < 0.01; compared with the HDM group, ^△△^ *p* < 0.01, *n* = 3.

Next, we evaluated the infiltration of inflammatory cells and the expression of Th-cell-related cytokines in the lung tissue of mice. We collected the BALF from mice and counted the total number of white blood cells (total cells), eosinophils (Eos), neutrophils (Neu), macrophages (Mac), and lymphocytes (Lym). As shown in Figure 3a, the number of total cells, Eos, Neu, Mac, and Lym in the OVA and HDM groups was considerably higher than that in the control group. Compared with the OVA or HDM groups, the number of these cells in the OVA + shikonin and HDM + shikonin groups was statistically reduced. Also, Th cell-related inflammatory factors in BALF were determined by ELISA. Compared with the control group, the expression of Th1-related cytokine IFN-γ in the OVA or HDM group was decreased, while the expression levels of pro-inflammatory cytokines IL-4 and IL-17 induced by Th2 cells and Th17 were increased. In addition, the expression of Treg-related cytokine IL-10 was declined. These results indicate that Th1/Th2 and Th17/Treg were imbalanced in asthma mice induced by OVA or HDM (Figure 3b). After shikonin treatment, the level of IFN-γ was increased, and Th2-related cytokine IL-4 and Th17-related cytokine IL-17 were down-regulated. In addition, shikonin up-regulated the level of IL-10 (Figure 3b). Consequently, shikonin can regulate the balance of Th1/Th2 and Th17/Treg in asthma mice. In summary, shikonin inhibits inflammation and relieves asthma by inhibiting lung inflammatory cell infiltration and regulating the expression of Th cell-related cytokines.

### 3.4 Effect of shikonin on the expression and localization of p-STAT3 in airway epithelial cells

**Figure 4:**
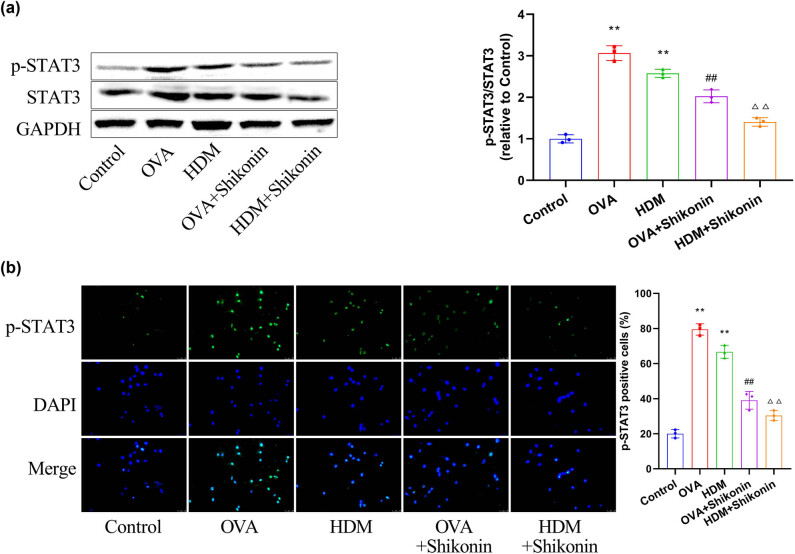
Shikonin suppresses the expression and localization of p-STAT3 in airway epithelial cells. (a) Western blot analysis was conducted to examine the phosphorylation level of STAT3 in mouse airway epithelial cells. (b) The expression pattern of p-STAT3 in airway epithelial cells was detected by immunofluorescence staining. Compared with the control group, ^**^ *p* < 0.01; compared with the OVA group, ^##^ *p* < 0.01; compared with the HDM group, ^△△^ *p* < 0.01, *n* = 3.

STAT3 plays an essential role in the production of inflammatory cytokines and is one of the important mechanisms leading to airway inflammation in asthma [26]. To explore whether shikonin alleviates asthma by regulating STAT3, we examined the activation of STAT3 and the nuclear translocation of p-STAT3. First, we compared the phosphorylation level of STAT3 in airway epithelial cells of mice in each group. Western blot results showed that the expression of p-STAT3 in the OVA or HDM group was notably higher than that in the control group. Protein levels of p-STAT3 were reduced in the OVA + shikonin and HDM + shikonin groups compared to the OVA or HDM group (Figure 4a). Next, we detected the expression pattern of p-STAT3 in airway epithelial cells by immunofluorescence staining. As shown in Figure 4b, the fluorescence intensity of p-STAT3 protein was significantly enhanced in the OVA and HDM groups, and the number of p-STAT3 positive staining cells was increased. However, treatment with shikonin decreased the number of p-STAT3-positive cells. These results indicate that shikonin can reduce the expression level of p-STAT3 and inhibit the transfer of p-STAT3 into the nucleus.

### 3.5 Activation of STAT3 reverses the inhibitory effect of shikonin on the function of MLE-12 cells

**Figure 5:**
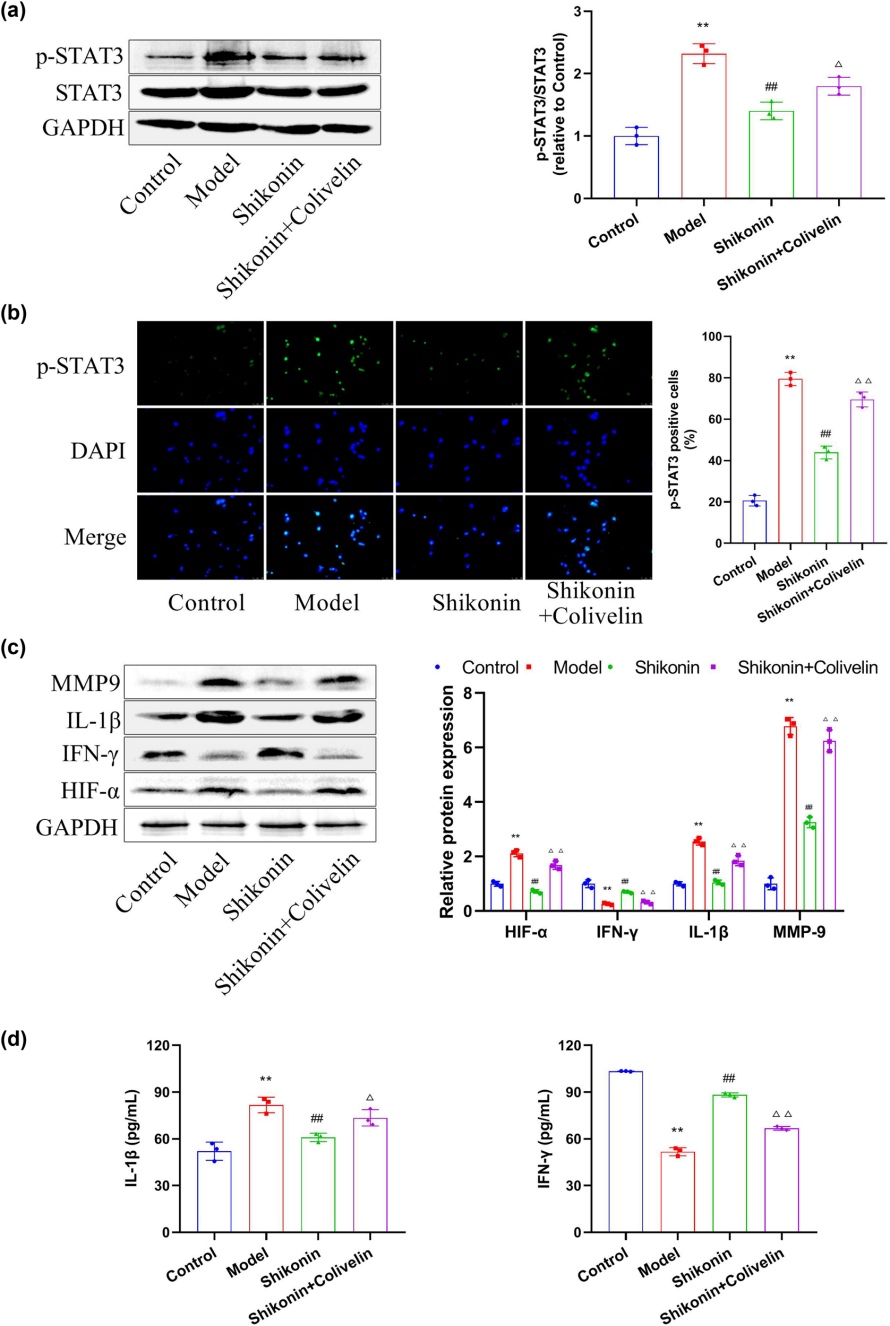
Activation of STAT3 can reverse the inhibitory effect of shikonin on the function of MLE-12 cells. (a) Western blot analysis was performed to measure the expression levels of STAT3 and p-STAT3 in mouse epithelial cell line MLE-12. (b) The localization of p-STAT3 in mouse bronchial epithelial cell line MLE-12 was detected by immunofluorescence staining. (c) Western blot was applied to measure the expression levels of HIF-α, IFN-γ, IL-1β, and MMP9. (d) ELISA was used to detect the concentrations of inflammatory factors (IL-1β and IFN-γ) in the MLE-12 cell medium. Compared with the control group, ^**^ *p* < 0.01; compared with the model group, ^##^ *p* < 0.01; compared with the shikonin group, ^△^ *p* < 0.05; compared with the shikonin group, ^△△^ *p* < 0.01, *n* = 3.

To further confirm the inference that shikonin can alleviate asthma by regulating STAT3, we observed the effect of shikonin on the expression and localization of p-STAT3 in TNF-α-induced mouse epithelial cell line MLE-12. In addition, MLE-12 cells were given 25 μg/mL colivelin (a STAT3 activator) in the presence of shikonin to observe whether activation of STAT3 could reverse the inhibitory effect of shikonin on asthma. As shown in Figure 5a, the expression of p-STAT3 was markedly increased under the action of TNF-α alone, which was inhibited by shikonin. Colivelin could reverse the inhibitory effect of shikonin on TNF-α-induced p-STAT3. Immunofluorescence staining showed that TNF-α promoted the transfer of p-STAT3 into the nucleus, while shikonin inhibited this phenomenon. Administration of colivelin in MLE-12 cells could antagonize this effect of shikonin (Figure 5b). Then, the expression of p-STAT3 downstream factors was detected by western blot in MLE-12 cells. Under the action of TNF-α, the expressions of the pro-inflammatory factor IL-1β were increased, the anti-inflammatory factor IFN-γ was decreased, and the levels of MMP9 and HIF-α were increased. After treatment with shikonin, the levels of IL-1β, MMP9, and HIF-α were inhibited and IFN-γ was up-regulated. However, the effects of shikonin on these factors were abated by colivelin (Figure 5c). The concentration of inflammatory factors (IL-1β and IFN-γ) in MLE-12 cell culture supernatant was detected by ELISA. Similar results were obtained (Figure 5d). In summary, activation of STAT3 can reverse the inhibitory effect of shikonin on the function of MLE-12 cells, confirming that the inhibitory effect of shikonin on asthma is dependent on STAT3.

## 4 Discussion

Recent studies have identified the therapeutic role of shikonin in a variety of autoimmune diseases such as systemic lupus erythematosus, psoriasis, inflammatory bowel disease, and rheumatoid arthritis [27]. Most importantly, intratracheal instillation of shikonin can down-regulate the release of IL-4, IL-5, IL-13, and TNF-α in BALF and reduce pulmonary eosinophils and airway hyperresponsiveness [28]. Based on the findings of this research, shikonin has emerged as a novel and promising therapeutic agent for the treatment of obstructive pulmonary diseases, including asthma. To further substantiate its potential use as an anti-asthmatic drug, we investigated the inhibitory effect of shikonin on asthma *in vivo* and *in vitro* and its underlying mechanism. The results demonstrate that shikonin effectively alleviates asthma symptoms in both experimental models and cell cultures. Mechanistically, the inhibitory effect of shikonin on asthma may be related to inhibiting the expression level of STAT3 in airway epithelium.

The prevalence of asthma characterized by chronic airway inflammation and airway hyperresponsiveness is still increasing [29]. Allergens play a pivotal role as external triggers for asthma [30]. HDM is an important interference factor affecting asthma treatment in indoor living environment, which can directly stimulate bronchial and airway epithelial cells to release pro-inflammatory cytokines and chemokines and induce direct, non-allergic inflammation, accompanied by elevated serum TH2-related immunoglobulin and cytokine levels [31,32,33,34]. OVA can induce both rapid and delayed allergic reactions, easily sensitizing individuals and causing airway inflammation, which is commonly used to study acute asthma triggered by food allergies [35]. To comprehensively investigate the therapeutic effect of shikonin on asthma, we utilized OVA and HDM to establish mouse models with different allergen-induced asthma phenotypes. The results demonstrated that shikonin effectively suppressed airway inflammation and hyperresponsiveness in mice with OVA or HDM-induced asthma.

Asthma is an inflammatory disease closely associated with immune imbalance-induced abnormal secretion of inflammatory factors involving Th1/Th2 and Th17/Treg [36,37,38]. Numerous studies have reported that the anti-inflammatory effects of shikonin primarily involve modulation of signaling pathways including MAPK, NF-κB, PKM2/STAT3, PI3K/AKT/mTOR, and Wnt/β-catenin [19,39,40,41,42]. Compared with previous reports, this study focuses on whether shikonin can restore the Th1/Th2 and Th17/Treg balance and regulate the production of inflammatory factors. Our results showed that shikonin reduced inflammatory cell infiltration. On the other hand, shikonin alleviated the imbalance of Th1/Th2 and Th17/Treg by regulating IFN-γ/FOXP3 and IL-4/IL-17A in asthma mice. Shikonin also exerted a regulatory effect on inflammatory factors such as IFN-γ, IL-4, IL-17A, and IL-10.

Airway epithelial STAT3 plays a key role in the occurrence of asthma, and its activation is associated with asthma symptoms such as airway hyperresponsiveness and lung and airway inflammation [24]. By studying whether shikonin affects asthma phenotype through STAT3, it was found that shikonin can reduce the expression level of p-STAT3 and inhibit the transfer of p-STAT3 into the nucleus in asthmatic mice. Moreover, shikonin lowered the TNF-α-induced increase of p-STAT3 in MLE-12 cells. An activator of STAT3, colivelin, reversed the TNF-α-induced increase of p-STAT3 and abrogated the effects of TNF-α on inflammatory factors in MLE-12 cells. This finding is consistent with our previous findings in lung adenocarcinoma cells [43]. In addition, disruption of Th17/Treg balance caused by activation of STAT3/RORγt-STAT5/Foxp3 signaling pathway aggravated WSI-TRPM2.5 and Oe-TRPM2.5-induced asthma exacerbation further supported our conclusion [44]. Importantly, targeting STAT3 for the treatment of asthma has achieved certain research findings. Direct targeting of the 3’UTR region of STAT3 by exosome miR-301a-3p reduced the release of inflammatory cytokines TNF-α, IL-1β, and IL-6, which can inhibit OVA-induced asthma [45]. α-Asarone inhibited the phosphorylation level of STAT3, thus inhibiting mast cell activation, inflammatory cell infiltration, and the release of IL-5 and IL-13 in lung tissue, and finally effectively alleviating allergic asthma [46]. However, how shikonin regulates the expression of p-STAT3 remains unclear. Zhang et al. have reported that in esophageal squamous cell carcinoma, down-regulation of p-STAT3 by shikonin is mediated by inhibition of p-PKM2 [47]. p-PKM2, the phosphorylated pyruvate kinase M2, is a key enzyme in cell metabolism and is involved in the regulation of glycolysis. Phosphorylated PKM2 can promote the activation of downstream NLRP3 and NF-κB, thereby leading to oxidative stress and inflammatory response [48]. However, whether p-PKM2 plays a role in asthma is unclear, and whether shikonin reduces p-STAT3’s abundance in airway epithelial cells through p-PKM2 needs further investigation, which will be the direction of our future research.

To sum up, our results show that shikonin alleviates the imbalance of Th1/Th2 and Th17/Treg in the airway epithelium of asthma mice and affects the release of inflammatory factors in MLE-12 cells through a STAT3-dependent mechanism. Our study provides a new evidence for shikonin as a potential anti-asthmatic drug.

## Abbreviations

OVA: ovalbumin
HDM: house dust mite
IL: interleukin
BALF: bronchoalveolar lavage fluid
Eos: eosinophils
Neu: neutrophils
Mac: macrophages
Lym: lymphocytes
STAT3: signal transducer and activator of transcription 3
TNF-α: tumor necrosis factor-α
IFN-γ: interferon-gamma
MAPK: mitogen-activated protein kinases
LPS: lipopolysaccharide
HE: hematoxylin/eosin
